# A prospective randomized controlled study of auricular point acupressure to manage chronic low back pain in older adults: study protocol

**DOI:** 10.1186/s13063-019-4016-x

**Published:** 2020-01-20

**Authors:** Chao Hsing Yeh, Cuicui Li, Ronald Glick, Elizabeth A. Schlenk, Kathryn Albers, Lorna Kwai-Ping Suen, Nada Lukkahatai, Nicole Salen, Sonaali Pandiri, Weixia Ma, Nancy Perrin, Natalia E. Morone, Paul J. Christo

**Affiliations:** 1 Johns Hopkins School of Nursing, 525 N. Wolfe Street, Room 421, Baltimore, MD 21205 USA; 2 Departments of Psychiatry, Physical Medicine and Rehabilitation, University of Pittsburgh School of Medicine, Pittsburgh, PA USA; 3 University of Pittsburgh School of Nursing, Pittsburgh, OH USA; 4 University of Pittsburgh School of Medicine, Pittsburgh, PA USA; 5 Hong Kong Polytechnic University School of Nursing, Kowloon, China; 6 Shandong Provincial Hospital Affiliated to Shandong University,, Jinan, China; 7 Boston University School of Medicine/Boston Medical Center, Boston, MA USA; 8 Johns Hopkins University School of Medicine, Baltimore, MD USA

**Keywords:** Chronic low back pain, Auricular point acupressure, Older adults, Cytokines

## Abstract

**Background:**

Chronic low back pain (cLBP) is a major health problem and the most common pain condition among those aged 60 years or older in the US. Despite the development of pharmacological and nonpharmacological interventions, cLBP outcomes have not improved and disability rates continue to rise. This study aims to test auricular point acupressure (APA) as a non-invasive, nonpharmacological self-management strategy to manage cLBP and to address current shortcomings of cLBP treatment.

**Methods/design:**

For this prospective randomized controlled study, participants will be randomly assigned to three groups: (1) *APA* group (active points related to cLBP), (2) Comparison group-1 (non-active points, unrelated to cLBP), and (3) Comparison group-2 (enhanced educational control, an educational booklet on cLBP will be given and the treatment used by participants for their cLBP will be recorded). The ecological momentary assessment smartphone app will be used to collect real-time cLBP outcomes and adherence to APA practice. Treatment and nonspecific psychological placebo effects will be measured via questionnaires for all participants. This proposed trial will evaluate the APA sustained effects for cLBP at 12-month follow-up. Monthly telephone follow-up will be used to collect study outcomes. Blood will be collected during study visits at baseline, post APA treatment, and follow-up study visits at 1, 3, 6, 9 and 12 months post completion of treatment for a total of seven assessments. Appointments will start between 9 and 11 am to control for circadian variation in cytokine levels.

**Discussion:**

This study is expected to provide vital information on the efficacy, sustainability, and underlying mechanism of APA on cLBP necessary for APA to gain acceptance from both healthcare providers and patients, which would provide a strong impetus for including APA as part of cLBP management in clinical and home settings.

**Trial registration:**

ClinicalTrials.gov, ID: NCT03589703. Registered on 22 May 2018.

## Background

Chronic low back pain (cLBP) is a major health problem and the most common pain condition among those aged 65 years or older in the United States (US) [1]. Despite the development of pharmacological and nonpharmacological interventions, cLBP outcomes have not improved and disability rates continue to rise [2]. Associated healthcare expenditure for cLBP is over US$253 billion annually, owing to medical care and disability-related productivity loss and wages [3]. Compared to those in middle-age, older adults have a higher prevalence of cLBP, with longer symptom duration, greater associated disability and depression [1, 4], and multiple medical conditions that require multiple medications.

Pharmacotherapy is the predominant treatment for chronic pain in current US medical practice for older adults. Since the 1980s, the prescription of opioids to treat chronic pain in the US has dramatically increased [5, 6]. Compared to younger adults, older adults account for a five-time increase in hospitalizations for opioid abuse [7]. This explosive increase in opioid use for chronic pain not only comes with significant risk, including misuse, overdose, and addiction [8], but also brings adverse side effects such as constipation, nausea, and somnolence [9–11], opioid-induced hyperalgesia [12, 13], and cognitive dysfunction [14, 15], Non-opioid pharmacotherapy is also associated with a variety of adverse side effects, such as drowsiness, constipation, dry mouth, gastrointestinal bleeding, liver and kidney toxicity, and addiction [16, 17]. Clearly, additional scalable, safe pain management approaches must be developed to provide patients with reliable, low-cost pain relief without new side effects, prolonged treatment, or frequent visits to healthcare providers.

We will test auricular point acupressure (APA) as a non-invasive, nonpharmacological self-management strategy to manage cLBP and to address current shortcomings of cLBP treatment. APA is derived from auricular acupuncture, which is an invasive (use of needles) and passive treatment (administered by a licensed practitioner). The therapeutic benefits of auricular acupuncture on pain have been recognized by the World Health Organization (WHO) [18]. In 1990, the WHO established a standardized, internationally accepted nomenclature of ear points and their locations [18]. Dissemination of auricular acupuncture is limited by the invasive acupuncture procedure with needles and by the few and small sample sizes of randomized clinical trials.

**Fig. 1:**
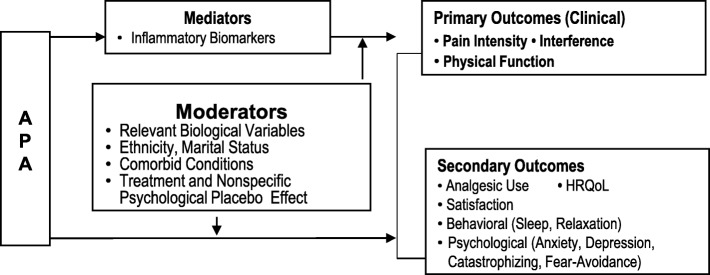
Framework of auricular point acupressure (APA) effects for chronic low back pain (cLBP)

*Theoretical framework* (Fig. 1). Grounded in the biopsychosocial model of pain [19–21], this study framework posits that the effects of APA on clinical, psychological, and behavioral outcomes for cLBP are mediated by inflammatory biomarkers and moderated by demographics, comorbid conditions (smoking status, obesity, and widespread pain symptoms) [22], and nonspecific psychological placebo effects (e.g., treatment beliefs [23–26], expectations [27, 28], and patient-provider relationships [29]). Evidence suggests that pain, anxiety [30, 31], depression [30, 32–35], catastrophizing [34, 36–40], and sleep [41] are interrelated, and that catastrophizing and fear-avoidance [36, 42–44] are highly associated with cLBP. A better understanding of these factors is critical to the development of more personalized and ultimately more effective approaches to managing cLBP. The National Institutes of Health Pain Consortium Research Task Force (NIH-Pain-RTF) has proposed a set of research standards to advance research on cLBP [22] to which this study will adhere. The primary outcomes involve the personal impact of cLBP (i.e., pain intensity, pain interference, and physical function). The secondary outcomes are analgesic use, anxiety, depression, fear avoidance, catastrophizing, sleep,, health-related quality of life (HRQoL), and treatment satisfaction. Comorbid conditions comprise smoking, obesity, and widespread pain symptoms [22]. Relevant biological variables comprise age, gender, and Body Mass Index (BMI). Other demographics include ethnicity and marital status. Nonspecific psychological placebo factors include treatment belief, treatment expectation, and patient-provider relationships.

## Methods/design

### Specific aims

Our specific aims are to (1) determine the efficacy of APA for relieving cLBP at 1 month after completion of APA (primary endpoint) and explore monthly follow-up to 12 months to assess sustained effects of APA; (2) determine the effects of APA on inflammatory signaling in cLBP; and (3) examine the relationships among potential mediators and moderators that may influence the beneficial effects of APA on cLBP.

#### Design

**Fig. 2:**
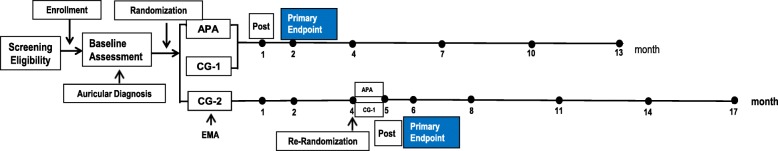
Overview of research design

**Fig. 3:**
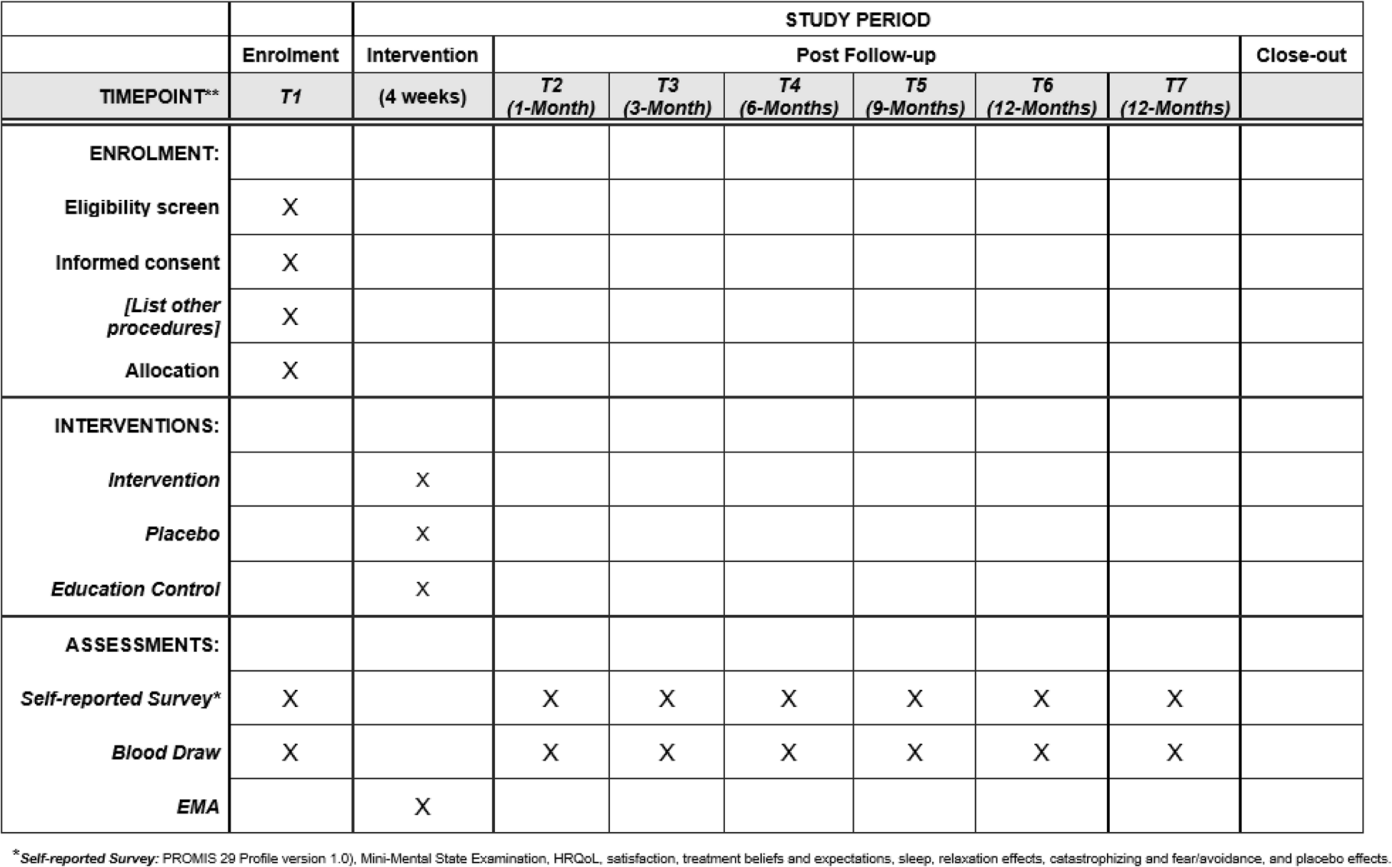
The schedule of enrollment, interventions, and assessments

For this prospective randomized controlled study, participants will be randomly assigned into three groups: (1) *APA* group (active points related to cLBP), (2) *Comparison group (CG)-1* (non-active points, unrelated to cLBP), and (3) *CG-2* (enhanced educational control, an educational booklet on cLBP will be given and the treatment used by participants for their cLBP will be recorded) (Fig. 2). Participants enrolled in CG-2 will be re-randomized into the APA group and CG-1 after 3-month follow-up assessment. For participants in the APA group or CG-1, blood will be collected during office visits at baseline, post APA treatment, and follow-up study visits at 1, 3, 6, 9 and 12 months post completion of treatment for a total of seven assessments (Fig. 2). For participants in CG-2, blood will be collected during study visits at baseline, post APA treatment, 1-month follow-up visit, as well as visits after re-randomization (i.e., baseline, post APA treatment, and follow-up offices visits at 1, 3, 6, 9 and 12 months post completion of treatment, for a total of 10 assessments (Fig. 3). The EMA (ecological momentary assessment) smartphone app will be used to collect real-time cLBP outcomes and adherence to APA practice. Treatment and nonspecific psychological placebo effects will be measured via questionnaires for all participants. Appointments will take place during the same time of day for each office visit to control for circadian variation in cytokine levels [45, 46]. All techniques have been established in our feasibility trial [47, 48]. Participants in CG-1will have the opportunity to receive APA after completion of the study assessment.

### Recruitment

Participants will be recruited through outpatient clinics at Johns Hopkins Hospital (JHH, including Johns Hopkins Pain Treatment Center, Johns Hopkins Community Physicians, Geriatric Medicine and General Internal Medicine Outpatient Clinics), the Community Engagement Program, social media (e.g., Craigslist), and the ClinicalTrials.gov website. We will use the registry of the Healthy Aging Studies Unit (the National Institute on Aging-funded Johns Hopkins Older Americans Independence Center Clinical Translation and Recruitment Core). The consent will be obtained by trained study coordinators.

### Study site

The study will be conducted at the Wald Community Nursing Center, Baltimore, MD, USA. The clinic has consultation rooms where face-to-face interaction with patients can occur. The clinic is staffed by nurses, nursing assistants, and reception staff.

For the study population inclusion and exclusion criteria, see Table 1.

**Table 1:** Study population inclusion and exclusion criteria

Inclusion criteria	Exclusion criteria
• Age 60 years or older • Able to read and write English • cLBP that has persisted for at least 3 months and causes pain on at least half of the days for the previous 6 months [22] • Average intensity of pain ≥ 4 on an 11-point numerical pain scale in the previous week • Have intact cognition (Mini-mental State Examination, MMSE score > 24) • Willing to commit to 4-weekly study visits and up to 12-months’ follow-up • Able to apply pressure to the acupressure seeds using tapes on their ears	• Malignant or autoimmune diseases (e.g., rheumatoid arthritis) • Known acute compression fractures caused by osteoporosis, spinal stenosis, spondylolysis, or spondylolisthesis because these conditions may confound treatment effects or the interpretation of results • Sciatica with leg pain greater than back pain • Allergy to the tape used • Use of some types of hearing aids (size may obstruct the placement of the acupressure seeds) • Pain in other parts of the body that is more severe than the cLBP and which occurs daily or almost every day with at least moderate intensity or acute pain; neurological disorders that could interfere with pain reporting or confound performance on the other outcomes, cerebral tumor, Alzheimer’s disease (or other cognitive illnesses), prior stroke, or multiple sclerosis *cLBP* chronic low back pain

**Table 2:** Summary of study measures

Construct	Specific measure	# Items	Reliability and validity	Timing
Screening				
	Mini-Mental State Examination		Good [50]	^b^
Primary outcomes: pain intensity, pain interference, physical function				
	minimal data set	4	Good [22]	^a, b^
Secondary outcomes				
Analgesic use	Quantification Score Version III		Objective [51]	^a, b^
HRQoL	Health-related quality of life	36	High [52]	^b^
Satisfaction	Treatment satisfaction	12	Good [47, 48]	4 weeks only
Psychological				
Anxiety, depression	Minimal data set	8	Good [22]	^b^
Fear/avoidance	Fear Avoidance Beliefs	16	Good [53]	^b^
Catastrophizing	Pain Catastrophizing Scale	13	Good [54]	^b^
Behavioral				
Sleep	Minimal data set		Good [22]	
Relaxation	Relaxation response	1	Pilot study	^b^
Moderating variables: comorbidity, demographics				
	minimal data set		Good [22]	Baseline
Nonspecific psychological placebo effects				
Expectations, beliefs	Treatment expectation	2	Good [47, 48]	Baseline
Relationship	Patient-provider relationship	7	Good [55]	^b^

^a^EMA, ecological momentary assessment; ^b^Baseline, post completion of treatment, monthly follow-up for 3 months

### Sample size justification/study power

The primary outcomes are measured at 1-month post completion of treatment. In our pilot study (i.e., pain intensity, pain interference, and physical function) [48], Cohen’s *d* (the effect sizes in terms of the standardized difference) ranged from 0.65 (physical function) to 1.28 (pain intensity) between the APA and the sham APA at 1 month after intervention. Our pilot sham group (CG-1, non-reactive points) should have similar or better outcomes than CG-2 (enhanced usual care). We assume that APA can sustain 90% effects at 12 months, the smallest effect size of physical function (0.65) would be 0.585 at 1-year follow-up. With a statistical power of 0.90 and an alpha level of 0.05, 63 participants per group are needed to detect significant differences between the APA group and either CG-1 or CG-2 using a repeated measures generalized linear model. We anticipate that approximately 30% of the enrolled participants will be lost to follow-up. Therefore, we will enroll 270 participants (90 per group). Due to the high prevalence of cLBP and our estimate of potential participants, we are confident to achieve this new sample size target of 270.

### Intervention: APA treatment protocol

The APA protocol follows the International Standards for Reporting Interventions in Clinical Trials of Acupuncture (STRICTA) guidelines [49]. For the APA group, ear points that will receive acupressure are within the two zones for cLBP located on the front and back of the ear and three points known for alleviating stress and pain (i.e., *shenmen, sympathetic*, and *nervous subcortex*). After the point is located, the interventionist will clean the outer ear, including the ear lobe, with 75% alcohol, then place pieces of pre-prepared tape with acupressure seeds on the participant’s ear. Points on both ears will be identified and used for treatment in the proposed study. This procedure will take 5–10 min. Participants will rest quietly in comfortable chairs during the process. To avoid bias, the interventionist will adhere to a scripted speech while interacting with participants. Participants will be instructed to contact the study center immediately if any of the pieces of tape fall off their ears for replacement or if adverse effects occur. From our pilot studies, we know that patients exhibit reduced cutaneous resistance for the low back zone [47, 48]. We have found that using three pieces of tape with two seeds each is needed to sufficiently cover the low back zone on the front and back of the ear.

#### Dose of treatment

Participants assigned to the APA group and CG-1 will be instructed to evenly press the tape and seeds covering each ear point without rubbing (to avoid skin damage and infection at the acupuncture point) for 3 min per time, three times daily (9 min total), even if they do not experience pain. A 2-s pause occurs between two tape pressings. The optimal pressure is considered to have been achieved when the participants feel localized tingling or mild discomfort. The tape and seeds will remain on ear points for 5 days. Participants will be instructed to remove both at the end of the fifth day. The interventionist will demonstrate the pressing technique to the participants, instructing them to apply steady pressure on the taped seeds until either mild discomfort or tingling is felt. Subsequently, the participants will do the pressing themselves. Patients will be instructed to contact the study center immediately and return for re-inspection, adjustment, and/or possible removal of the tapes if any adverse effects happen. The participants will also be instructed to not introduce any new medications or treatments for their pain during the time that they are in the intervention phase of the study. Once the study intervention phase has finished, the participants will be allowed to seek new methods of pain management as needed.

#### Frequency of treatment visits

The treatment duration will be 4 weeks with weekly cycles [48]. Each weekly cycle will include one office visit, 5 days of wearing the tape/seeds, and 2 days without, minimizing the risk of allergic reactions to the tape and allowing the ear points to recover and restore sensitivity prior to the next treatment. During each visit, the interventionist will place the tape/seeds on the ear points.

#### Intervention procedure

After auricular diagnosis, the principal investigator (PI) will write a treatment prescription (ear points for seed placement) for each participant’s APA in CG-1 by marking the points on each participant’s ear photo. The prescriptions will be saved as digital files labeled with the participant ID number, and given to the project coordinator, who will, give them to the interventionists to direct the seed placement after randomization.

### Comparison APA group (CG-1)

For the CG-1 group, the same procedure as for APA will be applied but the tapes/seeds will be placed on different points. Participants in the CG-1 will receive the APA on the five ear points, comprising *mouth, stomach, duodenum, internal ear,* and *tonsil* [47, 48]. These points are chosen for the sham APA treatment for two reasons. First, they are distinct from the zones of the ear (and the points therein) associated with the lower back, and correspond to body regions in which the participant is usually pain-free. Second, they are equivalent in number to those points used in the APA treatment group. Dose of treatment, frequency of treatment visits and intervention procedure are the same as for the APA treatment group.

### Enhanced educational control group (CG-2)

The CG-2 is essential for controlling possible placebo APA treatment effects, time, and attention. Therefore, the CG-2 will serve as the control group for any improvement over time, with education and interaction with study staff. Participants in the enhanced educational control group will be given the cLBP educational booklet published by the National Institutes of Health (https://www.niams.nih.gov/health-topics/back-pain) and visit the office weekly for assessment (i.e., blood draws and questionnaires), which is the same schedule as that for the APA group and CG-1. Participants enrolled in CG-2 will be re-randomized into the APA group and CG-1 at 3-month follow-up.

### Study measures

We will use the minimal data set to measure cLBP outcomes (PROMIS 29 Profile), which is recommended by NIH-Pain-RTF [22]. Participants can complete the data set within 7–10 min [22]. The Mini-mental State Examination [50] will be used to screen for cognitive function. We will also include measures of HRQoL, satisfaction, treatment beliefs and expectations, sleep, relaxation effects, catastrophizing and fear/avoidance, and placebo effects (Table 2). A paperless data-entry system, installed in iPads, will be used to allow data to be directly entered into the database.

#### EMA

*The ecological momentary assessment* **(**EMA) is a data collection method that is programmed in the smartphone app so that pain intensity data can be collected in real time. We have pilot-tested the smartphone app to collect data on participants’ adherence to the APA treatment and real-time pain outcomes [56, 57]. It was reported as easy to use and minimized recall bias [51]. Each participant will be provided with a smartphone and charger and instructed to use the EMA daily and charge the smartphone each evening. Our EMA app [56, 57] has been revised and customized to enhance user-friendliness, enlarged font-size screen, and adjust volume control to optimize use for older adults. The data collected through EMA will be used to calculate participants’ (1) frequency and duration of APA practice and (2) medication use and clinical outcomes (i.e., pain intensity, pain interference, and physical function), each measured by one question. The EMA app includes two surveys addressing: (1) random EMA of the real-time outcomes (pain intensity, pain interference, and physical function) and (2) time-contingent EMA for the adherence of APA practice and analgesic use, which will be prompted according to the participant’s schedule. The random EMA survey is programmed to deliver one random prompt per day during waking hours that are commensurate with each participant’s schedule. The four items addressing momentary pain/function level can be completed within 1 min. Time-contingent EMA survey entries of APA practice and analgesic use will take under 2 min to complete. Adherence will be calculated by the proportion of EMA completed and proportion of days in which APA practice goals are met. EMA questions will be presented one at a time on the screen. Participants will respond to each question by using the touch screen to move the cursor forward and back and then exit the questionnaire. EMA data will automatically upload to the project website in real time via an economical cell phone carrier data plan, and it will be evaluated every 72 h to determine usage. The smartphones selected are designed to support the Android or iPhone operating system separately and feature a large, high-resolution, color, liquid-crystal display (LCD) touch screen and non-volatile memory to avoid data loss in the event the battery discharges. Participants who do not enter EMA data for more than 2 days will be contacted to determine the reason, will be assisted in resolving any problems, and will then be encouraged to resume EMA recording.

#### Blood sample collection/testing for biomarkers

We will follow the protocol from our pilot studies for collecting and analyzing blood samples [58, 59]. A 15-mL blood sample will be drawn at baseline, 4-weekly office visits during APA treatment, and follow-up at 1, 3, 6, 9, and 12 months post completion of APA (10 time points). Blood samples will be collected using standard phlebotomy procedures and processed immediately for serum collection (coagulation and serum separation by centrifugation). Serum will be stored at − 80 °C at the Johns Hopkins University (JHU) School of Nursing. All specimens will be multiplexed and duplicated in assays and analyzed using Bio-Plex Manager software in the Immune Monitoring Core at the Johns Hopkins Oncology Center, which has extensive expertise in multiplex assays. The serum levels of interleukin (IL)-1α, IL-1β, IL-2, IL-4, IL-6, IL-8, IL-10, IL-12, IL-13, IL-17, interferon (IFN)-γ, tumor necrosis factor (TNF)-α, calcitonin gene-related peptide (CGRP), and transforming growth factor (TGF)-β will be measured using a multiplex, bead-based immunofluorescence assay performed by a blinded technician (Luminex-200 system, Version IS, Luminex, Austin, TX, USA). A five-parameter regression formula will be used to calculate the sample concentrations from the standard curves. The quantification of biomarkers will be performed in duplicate to verify the results. These assays typically exhibit high precision and reproducibility (i.e., 84.5% sensitivity, 98% specificity; 92% of the patients in the active disease group correctly classified from a cross-validation serum set) [60].

### Randomization

After baseline data have been collected, all eligible participants will be randomized into three groups. The randomization process will be performed by a systems analyst with the use of statistical software equipped with a random-number generator to create a list of group assignments before the study recruitment begins. This procedure will allow real-time randomization to occur immediately after baseline assessments. Randomization will occur in blocks of three or six, with assignments based on the specific number of expected eligible participants and divided equally between the three groups. The program locates the first unassigned record in the randomization list and assigns the participant to the group designated in that record. Participant identifier and date are written on the record.

### Blinding

*Participants* cannot be masked to the enhanced educational group to which they have been allocated because of the nature of the intervention. Participants in the APA group and CG-1 will be blinded regarding group assignment and will be evaluated for the treatment allocation after the first APA treatment, not at the end of the completed treatments to avoid bias due to the perceived treatment effects based on intelligent guessing. The *interventionists* will be blinded because (1) two interventionists will be trained; one for the APA group and one for CG-1 and (2) fidelity testing regularly implemented. The PI and co-investigators (Co-Is) will be blinded regarding group assignment and will not contact or interact with the participants during the intervention. The *data collector* for outcome assessments will be blinded since there will be no seeds placed on the ears when the data are collected. *Data analysis* personnel will be blinded because the information of the group assignment will be withheld and we will also use an independent statistician for analysis. Group allocation will be known by a minimum number of study personnel.

### Fidelity of intervention

Fidelity of the interventionists will be assured and assessed by four methods: (1) Interventionists will demonstrate their proficiency with written and oral examinations and will be observed and mentored during training; (2) The interventionists will take photos of every participant’s ear after the seeds have been placed and the photos will be sent to the PI for comparison to her treatment prescription to maintain at least 95% accuracy for the first 20 participants, and will then be randomly selected to check for accuracy monthly during the study. The inter-rater reliability (Kappa) of point identification accuracy between the PI and the interventionists will be established. If Kappa scores are less than 0.8, further training will occur; (3) The PI will develop a schedule of random visits with interventionists to observe their adherence to protocol in real-time; and (4) To avoid any bias caused by the interventionists, they will be taught to adhere to a scripted speech during interaction with participants.

### Data management

We will use a paperless data-entry system with password-protected access to allow data to be entered directly into the database. All measures for office visits will be in electronic form with customized data entry, and a direct data-entry system will be installed on iPads. Data will be collected during one-on-one sessions with participants, eliminating the need for and cost of double data entry using paper forms, and decreasing missing and incorrect data entries. All of the data and uploaded EMA data will be encrypted and stored in a Microsoft SQL server on the JHU project website with a date/time stamp. All participants will be assigned unique study identifiers that will appear on all data collection instruments, documents, and files used in statistical analysis and manuscript preparation. Personal information is needed for tracking informed consent, which will be stored separately from other data and accessible only to select team members. No participant’s information will be released. Serum for biomarker analysis will be labeled with study ID and date of phlebotomy and kept at − 80 °C in a locked room.

### Treatment of missing data

With the use of direct data entry, we expect to minimize missing data. We will obtain reasons for study dropout so that we can assess the missing data mechanism. If the assumption of missing at random (MAR) is reasonable, likelihood-based methods that ignore the response mechanism will be appropriate [61]. If the probability of missingness depends on the unobserved data even after the observed data are conditioned (i.e., non-ignorable missingness), we will consider both selection models and pattern-mixture models to guide our chosen model [61]. The effect of our assumptions on inferences regarding the missing mechanism will be assessed by using sensitivity analyses [62].

### Data analysis plan

Data will be audited by an independent safety officer annually and analyzed in phases, with initial screening of data followed by primary analysis to address each research aim. The *intent-to-treat* approach, with inclusion of all participants randomly assigned to groups— regardless of adherence, treatment received, or withdrawal—will be used to address our specific aims [63–65]. Adherence to the assigned group will be monitored and examined in exploratory analyses. All statistical analyses will be preceded by detailed descriptive summaries. Violations of assumptions underlying planned methods will be checked, and appropriate data transformation will be performed, if needed. Data analyses will be conducted using Statistical Analysis System (Statistical Analysis System (SAS) Institute Inc., Cary, NC, USA).

In order to examine Aim 1 to determine the efficacy of APA for cLBP at 1 month post treatment, we will use a general linear mixed model (GLM) to construct a multilevel model for comparing the differences in the change in the outcomes from baseline to 1 month among the three groups. The group-by-time interaction is the main parameter of interest. For Aim 2 to evaluate the effect of APA on biomarkers, the analytical strategies for Specific Aim 1 will be used. Outcomes at 1 month post treatment will be examined using each biomarker individually and two latent variables with pre-defined biomarkers within the groups of pro- and anti-inflammatory biomarkers. For Aim 3 to examine whether or not demographics, comorbid conditions, and placebo effects moderate the relationship between APA treatment on primary and secondary outcomes, the previously described regression models in Specific Aim 1, which include the main effect terms for the intervention or group, will be expanded to include the main effect terms for moderators and the three-way interaction terms between group, time, and moderators. Data from the smartphone EMA app will be analyzed per our published methods and the following analysis plan [57, 66]. Adherence to APA practice, medication use, and real-time cLBP outcomes will be calculated and compared among groups using a multivariate analysis of variance (MANOVA) or a chi-square test to compare the differences between groups over time. Joinpoint regression [61] will be used to estimate the linear trend of improvement in percentages of outcome score over time. This strategy will help to analyze trends with different lines connected together at some joinpoints based on a log linear regression:

$$ \mathit{\ln}\ \left(\mathrm{percentage}\right)={\beta}_0+{\beta}_1\times \left(\mathrm{calendar}\ \mathrm{day}\right). $$

Each joinpoint represents a significant change in the trend slope; the best number of joinpoints will be determined by a permutation test.

## Discussion

There are some potential challenges for this study. Adherence is one of the challenges. EMA data will be checked every 72 h to determine usage. Participants who fail to respond to EMA prompts for more than 2 days will be contacted to determine the reason for not doing so. Fidelity of APA is also a challenge**.** Ear photos after seed placement will be taken and will be evaluated by the PI’s marked photos to ensure competency to administer APA with fidelity. Further training will be provided if necessary. We are aware that reducing participant burden is important.

Other challenges include recruitment and dropout**.** Recruitment will be monitored closely. We have the support of a cohesive team of experienced Co-Is who are recognized in their areas of knowledge and will contribute their complementary expertise to this project. Changes in recruitment procedure will be made if lower-than-expected recruitment occurs. The changes to the protocol are waiting to be approved by the IRB. We will provide participants with free parking or transportation to facilitate coming to sessions, and reimburse them for their time. We will also call/reschedule when a treatment session is missed. Participants in the CGs will have opportunities to receive APA after they complete the study. For participants who decline further treatment or are unable to come in for follow-up assessments, we will contact them to complete questionnaire data by telephone. Although we did not have complaints of excessive burden in the pilot study and observed a 90% retention rate after receiving the first APA treatment, we have further minimized burden with the smartphone to collect the daily data.

This study will provide vital information on the efficacy, sustainability, and underlying mechanism of APA on cLBP necessary for APA to gain acceptance from both healthcare providers and patients, which would provide a strong impetus for including APA as part of cLBP management in clinical and home settings.

### Trial status and publication plan

Protocol version number and date: NCT03320200, July 2018. Recruitment began in March 2019. Recruitment will be completed in February 2023. After recruitment completion, data will be analyzed and published in peer-review journals or presentations at professional conferences, then the final report will be prepared and published.

## Supplementary information

**Additional file 1.** SPIRIT checklist.

## Data Availability

Not applicable

## Abbreviations

APA: Auricular point acupressure
BMI: Body Mass Index
CG: Comparison group
CGRP: Calcitonin gene-related peptide
cLBP: Chronic low back pain
Co-I: Co-investigator
EMA: Ecological momentary assessment
GLM: General linear mixed model
HRQoL: Health-related quality of life
JHH: Johns Hopkins Hospital
JHU: Johns Hopkins University
LCD: Liquid-crystal display
MAR: Missing at random
NIH-Pain-RTF: National Institutes of Health Pain Consortium Research Task Force
PI: Principal investigator
SAS: Statistical Analysis System
STRICTA: Standards for Reporting Interventions in Clinical Trials of Acupuncture
US: United States
WHO: World Health Organization

## Availability of data and materials

The data sets analyzed will be available from the corresponding author on request.
